# In vivo vaccination with cell line-derived whole tumor lysates: neoantigen quality, not quantity matters

**DOI:** 10.1186/s12967-020-02570-y

**Published:** 2020-10-21

**Authors:** Inken Salewski, Yvonne Saara Gladbach, Steffen Kuntoff, Nina Irmscher, Olga Hahn, Christian Junghanss, Claudia Maletzki

**Affiliations:** 1 Department of Medicine, Clinic III-Hematology, Oncology, Palliative Care, Rostock University Medical Center, Ernst-Heydemann-Str. 6, 18057 Rostock, Germany; 2 Institute for Biostatistics and Informatics in Medicine and Ageing Research (IBIMA), Rostock University Medical Center, University of Rostock, 18057 Rostock, Germany; 3 Faculty of Biosciences, Heidelberg University, 69120 Heidelberg, Germany; 4 Division of Applied Bioinformatics, German Cancer Research Center (DKFZ) and National Center for Tumor Diseases (NCT), Heidelberg, Germany; 5 Department of Cell Biology, Rostock University Medical Center, 18057 Rostock, Germany

**Keywords:** Tumor lysate, Mutational load, MMR deficiency, In vivo imaging, Primary cell lines

## Abstract

**Background:**

Cancer vaccines provide a complex source of neoantigens. Still, increasing evidence reveals that the neoantigen quality rather than the quantity is predictive for treatment outcome.

**Methods:**

Using the preclinical Mlh1^−/−^ tumor model, we performed a *side-by side* comparison of two autologous cell-line derived tumor lysates (namely 328 and A7450 T1 M1) harboring different tumor mutational burden (TMB; i.e. ultra-high: 328; moderate-high: A7450 T1 M1). Mice received repetitive prophylactic or therapeutic applications of the vaccine. Tumor incidence, immune responses and tumor microenvironment was examined.

**Results:**

Both tumor cell lysates delayed tumor formation in the prophylactic setting, with the A7450 T1 M1 lysate being more effective in decelerating tumor growth than the 328 lysate (median overall survival: 37 vs. 25 weeks). Comparable results were achieved in therapeutic setting and could be traced back to antigen-driven immune stimulation. Reactive T cells isolated from A7450 T1 M1-treated mice recognized autologous Mlh1^−/−^ tumor cells in IFNγ ELISpot, but likewise YAC-1 cells, indicative for stimulation of both arms of the immune system. By deciphering local effects, vaccines shaped the tumor microenvironment differently. While A7450 T1 M1 prophylactically vaccinated tumors harbored low numbers of myeloid-derived suppressor cells (MDSC) and elevated CD8-T cell infiltrates, vaccination with the 328 lysate evoked MDSC infiltration. Similar effects were seen in the therapeutic setting with stable disease induction only upon A7450 T1 M1 vaccination. Untangling individual response profiles revealed strong infiltration with LAG3^+^ and PD-L1^+^ immune cells when treatments failed, but almost complete exclusion of checkpoint-expressing lymphocytes in long-term survivors.

**Conclusions:**

By applying two tumor cell lysates we demonstrate that neoantigen quality outranks quantity. This should be considered prior to designing cancer vaccine-based combination approaches.

## Background

The idea of using whole tumor lysates as vaccines dates back to the late 1970ies and aims at the induction of a vigorous immune response against cancer [1]. Highly immunogenic tumor-derived neo-epitopes must be present to be recognized by cytotoxic T cells. Antigen (Ag)-loaded dendritic cells (DCs) are the most commonly used cell-based vaccines with proven safety and, notably, the capability of providing long-lasting protective immunity [2–4]. As such, vaccines hold promise to delay or prevent cancer recurrence, particularly in early-stage disease patients, when immune-suppressive mechanisms are not firmly established. They conquer the limitations of classical peptide-based approaches by not creating favorable conditions for growth of tumor cell clones that lack the Ags present in the vaccine [5]. Still, therapeutic cancer vaccines have met limited clinical success [6, 7]. In most cases, the immune system is either polarized and/or has a limited tumor-specific T cell repertoire [8, 9].

Several strategies were employed to prepare Ags from whole tumor cells and thus produce a highly immunogenic vaccine. Common strategies include chemical treatment, radiation as well as repetitive freeze/thaw cycles. With these methods, standardized, applicable sources of tumor-specific Ags can be generated. Besides, tumor cell lysates are also useful in high-risk, tumor-free patients—especially for prophylactic approaches.

Lynch syndrome (LS), the most common hereditary cancer syndrome, represents the paragon for cancer vaccination approaches [10–12]. Affected patients suffer from a deleterious germline mutation in one of the mismatch repair genes (MMR) and develop a complex spectrum of solid cancers [13–15]. Having in mind that almost all tumors in LS patients are hypermutated and microsatellite instable (MSI), they are likely to express a huge amount of neo-Ags. These, in turn, may elicit an Ag-specific cytotoxic T-cell response [16].

To move forward in developing vaccination strategies, we employed the MLH1^−/−^ mouse model that resembles features of the human LS counterpart [17, 18]. These mice develop spontaneous tumors at virtually 100% frequency [18] and are suitable for prophylactic as well as therapeutic approaches. Indeed, in our previous studies, we vaccinated mice with an allograft-derived whole tumor lysate [19, 20]. While this approach proved successful, direct transfer into the clinic might be compromised by the fact that tumor lysate preparation is only applicable for previously diseased patients with a high likelihood of relapse. Another critical and limiting factor is the amount of the original material and the timely delivery of the individually tailored vaccine. Hence, we addressed the question of whether cell-line derived tumor lysates might provide an alternative source of highly immunogenic tumor Ags. In a pilot study, we identified different outcomes upon vaccination with two individual cell line-derived lysates in the therapeutic setting [21]. We hypothesized that the mutational signature predicts response. Here, we refined our cell line-tailored vaccination approach aiming at a detailed understanding of the mechanisms underlying vaccination efficacy. Our results show that tumor cell lysates delay tumor formation and growth; still, the neo-Ag quality rather than the quantity is predictive for response.

## Material and methods

### Cell culture and tumor lysate preparation

Mlh1^−/−^ cells (two gastrointestinal tumor (GIT) cell lines: 328, A7450 T1 M1 and one lymphoma cell lines: 1351) were established in our lab. YAC-1 cells were originally cultured in DMEM medium, supplemented with 10% FCS (fetal calf serum), 6 mM Glutamine, and penicillin/streptomycin antibiotics (all from Biochrom, Berlin, Germany). Tumor lysates were prepared from cell cultures in P15 as described [20]. Briefly, confluent cells were harvested and treated with four repetitive freeze/thaw cycles followed by 60 Gy irradiation and protein quantification. Lysate stocks were frozen at − 80 °C and used for in vivo application.

### DC generation and co-culture

DCs were generated from murine femur and tibia as described [22]. Briefly, the resulting cell suspension was filtered (100 μm, Greiner-bio one, Kremsmünster, Austria) and centrifuged at 300×*g* (10 min, 4 °C). Cells were seeded in a 6-well plate (density: 3 × 10^5^ cells/ml). GM-CSF was added (20 ng/ml, Immunotools, Friesoythe, Germany) cells were harvested every third day. Therefore, non-adherent cells were gently pipetted up and down, transferred in a centrifuge tube, pelleted (200×*g*, 8 min), the supernatant discarded and the pellet resuspended in freshly prepared medium. Cells were counted and re-cultured in DC medium containing GM-CSF with no other cytokines to generate highly pure DCs. On the 9th day, supernatant was collected and centrifuged. DCs were phenotyped using the following FITC-, PE-, APC-, and PE/Cy7-labeled antibodies (1 μg each): anti-CD11c (Biolegend, San Diego, CA), anti-CD83 (Biolegend), anti-CD11b (Immunotools), anti-CD40 (Biolegend), anti-CD80 (Immunotools), anti-CD86 (Immunotools), anti-MHC class I/II (Immunotools), and anti-CD19 (Immunotools). Afterwards, DCs were loaded with protein lysate (50 µg/tumor lysate). After 24 h of incubation, peripheral blood mononuclear cells were added in a ratio of 1:10 (DC:immune cell) [23, 24] and a co-culture was established. On the 5th day, Brefeldin A (5.0 µg/ml Biolegend) was used to enhance intracellular cytokine staining signals. The following fluorescent-labeled antibodies (1 μg each) were used: anti-CD3, anti-CD4, anti-CD8α, anti-CD25 (Immunotools), anti-IFN-γ, and anti-TNF-α (Biolegend). Immunophenotypic changes were determined using flow cytometry (BD FACSVerse™, BD Pharmingen, Heidelberg, Germany).

### Visualization of whole exome sequencing data

The cell lines 328 and A7450 T1 M1 were processed likewise [20, 21] for the visualization. With the complex Heatmap [25] R package, their patterns and correlations were revealed in oncoprint. The mutational profiles were filtered for the exclusive SNV separately with mutation filters such as mutation type (missense and nonsense) and those occurring in known annotated genes.

Furthermore, mapping the mutations and their statistics on a linear gene product (proteins of interest) was done with a ‘lollipop’ mutation diagram generator [30]. Based on the knowledge from the human MMR-D counterpart and general involvement in tumorigenesis, genes for further analysis were chosen with a high probability of mutating.

### Mlh1^−/−^ mouse model and in vivo vaccination protocol

#### Ethical statement

All animal experiments were approved by the German local authority: Landesamt für Landwirtschaft, Lebensmittelsicherheit und Fischerei Mecklenburg‐Vorpommern (7221.3‐1‐026/17), under the German animal protection law and the EU Guideline 2010/63/EU. Mice were bred in the animal facility of the University Medical Center in Rostock under specific pathogen‐free conditions. Mlh1 genotyping was done according to [26]. During their whole life-time, all animals received enrichment in the form of mouse-igloos (ANT Tierhaltungsbedarf, Buxtehude, Germany), nesting material (shredded tissue paper, Verbandmittel GmbH, Frankenberg, Deutschland) paper roles (75 × 38 mm, H 0528–151, ssniff‐Spezialdiäten GmbH, Soest, Germany), and wooden sticks (40 × 16 × 10 mm, Abedd, Vienna, Austria). During the experiment, mice were kept in type III cages (Zoonlab GmbH, Castrop‐Rauxel, Germany) at 12‐h dark:light cycle, the temperature of 21 ± 2 °C, and relative humidity of 60 ± 20% with food (pellets, 10 mm, ssniff‐Spezialdiäten GmbH, Soest, Germany) and tap water ad libitum.

#### Experimental protocol

**Fig. 1:**
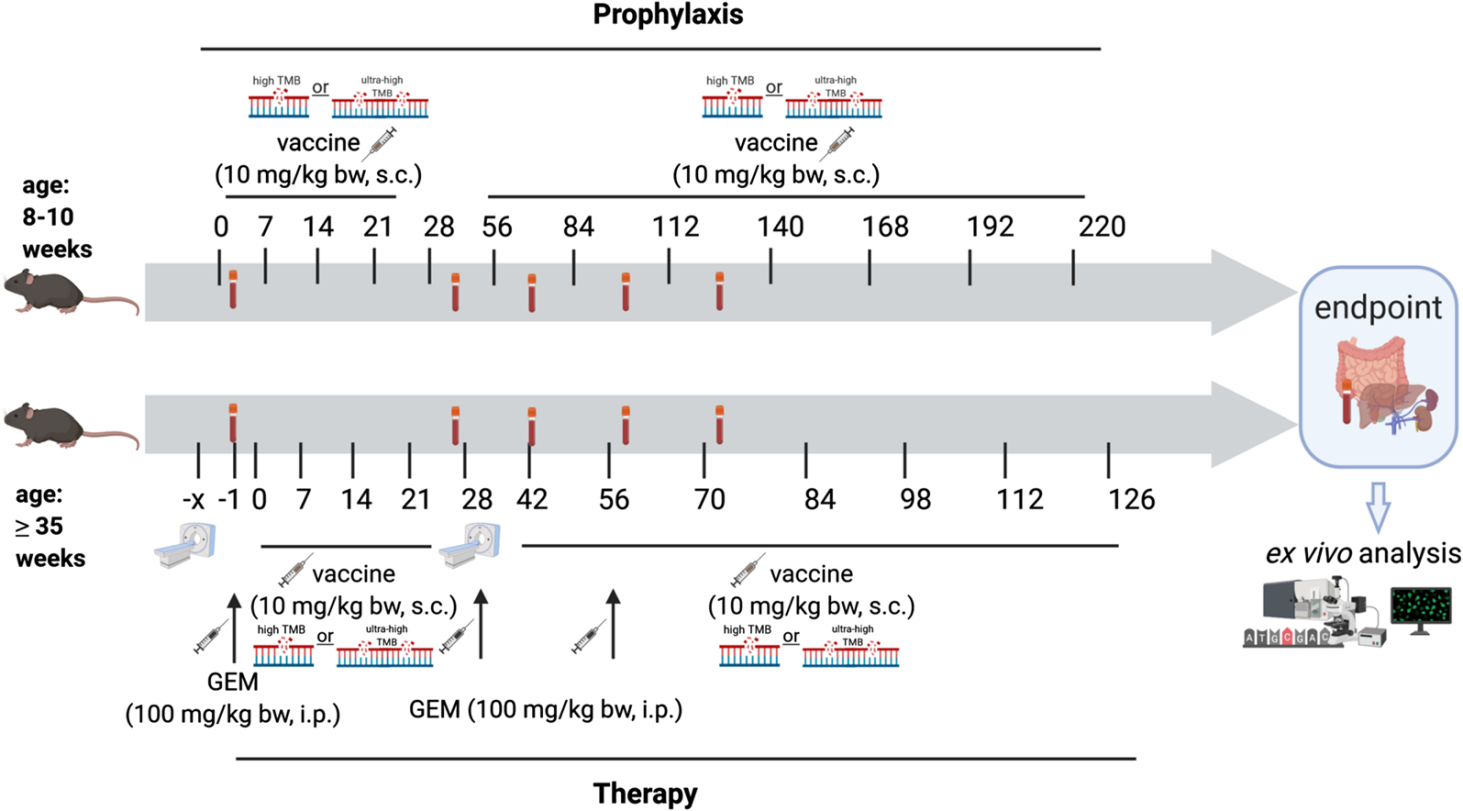
Vaccination protocol. Mice were either given repetitive prophylactic or therapeutic injections of two different cell line-derived whole tumor lysates. In the case of therapeutic application, mice were additionally given 3 monthly injections of gemcitabine. The scheme was created with BioRender.com

A detailed treatment schedule is provided in Fig. 1. Briefly, prophylactic application was initiated when mice aged 8–10 weeks by four weekly boosts of tumor lysates (10 mg/kg bw, s.c., 328 vaccine: n = 10; A7450 T1 M1 vaccine: n = 9, respectively) followed by monthly applications (a total of 12 vaccinations). Control mice were left untreated (n = 15 mice). For the therapeutic vaccination approach, mice were given 4 weekly boosts. Vaccination was sustained (10 mg/kg bw, biweekly) until tumors progressed (max. 12 injections; n = 8 mice/group). Control mice were left untreated (n = 7 mice). Reduction of suffering during the trial was guaranteed by providing daily prepared soaked pellets, twice-daily monitoring of the health status using a score sheet and by applying humane endpoints (weight loss, any sign of pain or distress, or changes in social behavior). All mice were sacrificed before they became moribund to prevent pain and distress.

### Positron emission tomography/computed tomography (PET/CT) imaging

PET/CT imaging scans were performed on a small animal PET/CT scanner (Inveon PET/CT, Siemens Medical Solutions, Knoxville, TN, USA) according to a standard protocol as described before [19, 20].

### Immune phenotyping and immunofluorescence

Blood samples were taken routinely from the retrobulbar venous plexus. Blood samples were stained with a panel of conjugated monoclonal antibodies (mAb, 1 μg each) followed by lysis of erythrocytes (155 mM NH_4_Cl, 10 mM KHCO_3_ (both MERCK Millipore, Darmstadt, Germany), and 0.1 mM EDTA (Applichem, Darmstadt, Germany). Negative controls consisted of lymphocytes stained with appropriate isotypes (Biolegend, San Diego, USA). Cells were washed, resuspended in PBS and analyzed by flow cytometry on a Flow Cytometer (BD FACSVerse™, BD Pharmingen, CA, USA). Data analysis was performed using BD FACSuite software (BD Pharmingen).

Target proteins in 4 µM cryostat sections of tumor resection specimens were visualized as described [20] and documented on a confocal laser scanning microscope (LSM780, Zeiss, Jena, Germany) using 20× objectives.

### Procartaplex Cytokine Assay

Cytokine levels in cell culture supernatants as well as plasma samples were determined according to the manufacturer’s instructions of the Procartaplex™ multiplex immunoassay. Measurement as well as cytokine quantification was performed on a Bioplex 2000 (Bio-Rad Laboratories GmbH, Munich, Germany) in combination with the Bio-Plex Manager Software.

### IFNγ ELISpot

2.5 × 10^3^ targets/well (Mlh1^−/−^ A7450, Mlh1^−/−^ 328, Mlh1^−/−^ 1351, and YAC-1 cells) were seeded in IFNγ–specific mAb (Mabtech, 3321-3)-coated, 96-well microtiter plates. Peripheral blood leukocytes (5 × 10^4^ cells/well) or splenocytes (1 × 10^4^ cells/well) were added in triplicates and co-cultured overnight. Finally, bound antibody (Mabtech, 3321-6) was visualized by BCIP/NBT (KPL, Gaithersburg, Maryland, USA); spots were counted using an ELISpot reader. Presented are the numbers of IFN*γ*-secreting cells corrected for background levels counted in the absence of target cells, which was always ≤ 5 spots/well. Target cells without effector cells showed no background level.

### Statistics

All values are expressed as mean ± SD. In case of PET/CT data, raw tumor sizes are presented. After proving the assumption of normality (Kolmogorov–Smirnov test), differences between vaccinated and control mice were determined using the unpaired Student’s t-test or one-way ANOVA (Bonferroni or Dunnett’s multiple comparison). Kaplan–Meier survival analysis was done by applying the log rank (Mantel Cox) test. Statistical analyses were performed using GraphPad Prism 5 (San Diego, CA). The criterion for significance was set to p < 0.05.

## Results

### In vitro characterization of antigen-sources

**Fig. 2:**
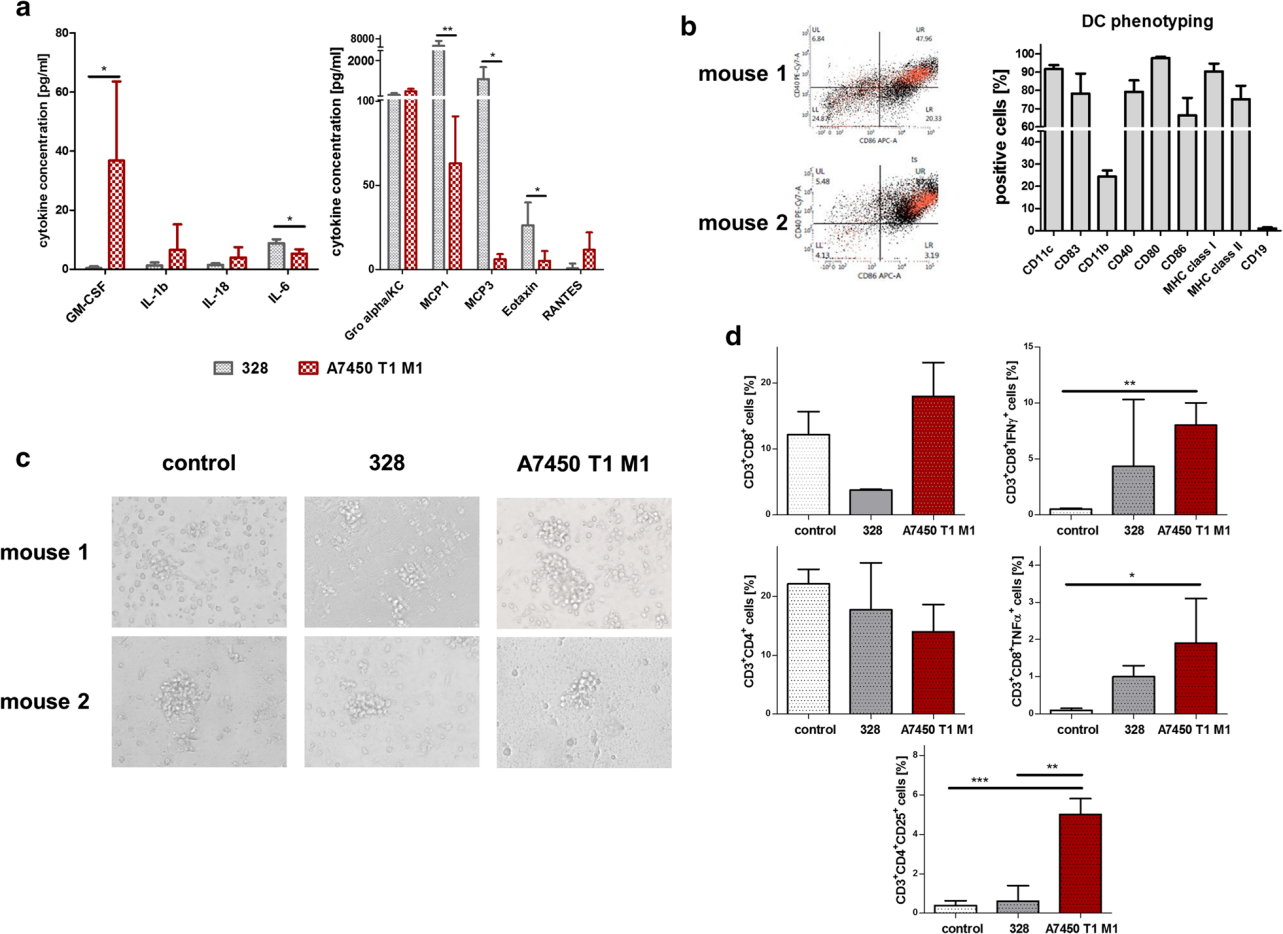
Secretion profile and in vitro DC culture. **a** Cell culture supernatants were collected and analyzed using the Procartaplex™ multiplex immunoassay. Finally, cytokine and chemokine concentration were determined from three independent experiments. Given are the mean + SD. *p < 0.05; **p < 0.01; unpaired one-sided T-test. **b** Left panel: representative flow cytometry of DCs and right panel: quantitative phenotyping of DCs taken from three individual mice. Non-adherent cells were analyzed. Given are the % numbers of positive cells measured upon gating on viable cells (n  =  3 mice). **c** Representative microscopic images of DCs loaded with tumor A7450 T1 M1 or 328 tumor lysate, respectively. Original magnification 10×. **d** Phenotyping of peripheral blood mononuclear cells after co-culture with loaded DCs (DC:immune cell ratio: 1:10). On the 5th day, Brefeldin A was used to enhance intracellular cytokine staining signals. Immunophenotypic changes were determined using flow cytometry, data analyses were performed using BD FACSuite software. Given are the % numbers of positive cells measured upon gating on viable cells. *p < 0.05; **p < 0.01; ***p < 0.001; one-way ANOVA (Bonferroni multiple comparison)

In this study, two Mlh1^−/−^ cell lines established from spontaneous GIT were used. The drug response of the cell lines 328 and A7450 T1 M1 was determined before and revealed no significant differences towards standard cytostatic drugs [19]. By assessing the basal secretion profile from supernatants, we indeed observed substantial variations. Focusing on cytokines associated with immune stimulation, A7450 cells generally secreted higher levels of GM-CSF, IL1b, and IL-18 (Fig. 2a, left panel). While all of these cytokines enhance NK cell activity and foster Th1 cell development, concentration of chemokines responsible for monocyte- and eosinophil-attraction, such as monocyte chemoattractant protein-1(MCP1), MCP3, and Eotaxin was higher in supernatants from 328 cells (Fig. 2a, right panel).

Based on these findings, a co-culture system of tumor-Ags-loaded DCs and lymphocytes was initiated. DCs were established from the bone marrow according to a standard protocol using GM-CSF [22]. We decided to use this method for DC generation because it delivers highly pure DCs (> 90% purity), constituting a mixture of immature and mature DCs (Fig. 2b). By flow cytometry, virtually all cells expressed DC-markers CD11c, CD83 as well as co-stimulatory molecules CD80/86. CD11b was reduced, mainly because of their activation status (Fig. 2b). In the co-culture setting, additional differences were seen in the T cell phenotype (Fig. 2c, d). DC-loaded with A7450 T1 M1 tumor lysate boosted the frequency of CD3^+^CD8^+^ T cells, which were activated and additionally positive for IFNγ. By contrast, the phenotypes of leukocytes from 328 lysate-loaded DCs changed faintly compared to the control (Fig. 2b).

### Mutational profile of antigen-sources

**Fig. 3:**
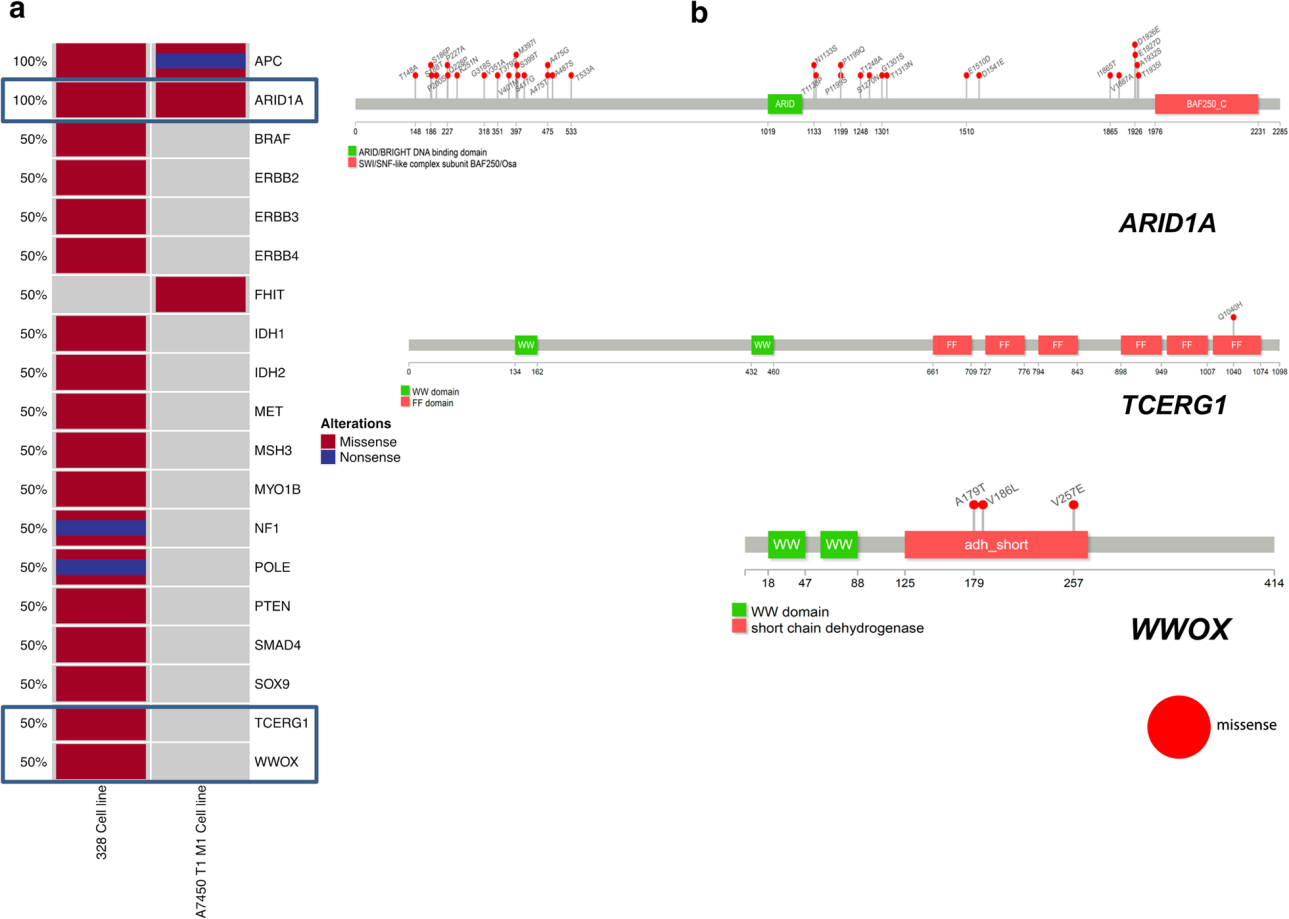
Human counterpart hotspots in cell line development. **a** The presented oncoprint reveals patterns and correlations of the different cell lines 328 and A7450 T1 M1 with regard to tumor suppressive functions, tumor initiation, and progression. An overview of the non-synonymous alterations in genes of interest (rows) affecting the cell lines (columns) is provided with this way of visualization. **b** Prevalence and hotspot regions in *Arid1a*, *Tcerg1*, and *Wwox*. The colored regions show known gene/ protein domains, while the other regions are represented in dark grey. The missense mutations are depicted as a red lollipop in the cell lines, whereby each lollipop label shows an amino acid change with its corresponding location

The selected genes of the oncoprint are known for the relevance for tumor initiation, progression, apoptosis, and suppressors functions (Fig. 3a). Mlh1^−/−^ tumors harbor mutations in *Pik3ca*, *Msh3*, *Braf*, and/or *Kras*, and *Erbb3* [21]. The A7450 T1 M1 cell line harbors nonsense and missense single nucleotide variants (SNVs) in the *Wnt* signaling pathway regulator *Apc* gene. Further hotspots in pre-selected clinical relevant genes are occurring in tumor suppressors *Arid1a* as well as *Fhit*.

In a direct comparison, alterations are exclusively distributed. The cell line 328 acquired more missense SNVs in the pre-selected gene set, especially in EGFR signaling members as well as in *Nf1*. The 328 cell line had additional missense and nonsense *Pole* mutations. Taking the germline MMR-D into account, the increased number of gene mutations in affected tumor cells is conserved in the cell line 328 compared to A7450 T1 M1 (Fig. 3a).

In *Arid1a*, all of the 34 SNVs appear before or after the ARID/BRIGHT DNA binding domain (Fig. 3b), regulating cell proliferation, differentiation, and development [27], as well as the SWI/SNF-like complex subunit BAF250/Osa. Every single SNV is exclusive for the corresponding cell line, none are shared, and all of them are missense mutations.

The prevalence and hotspot mutations in *Tcerg1* and *Wwox* exclusively detected in the 328 cell line are shown in Fig. 3b. The mutational hotspot in *Tcerg1* is Q1040H within the FF6 domain, the only amino acid change in this gene. FF domains play an essential role in binding the phosphorylated C-terminus of the RNA polymerase II. Furthermore, *Tcerg1* is involved in regulating the transcriptional elongation and the pre-mRNA splicing [28]. In *Wwox*, we found three SNVs, which all affect the short-chain of the dehydrogenase/reductase domain.

For the mutational profile as a potential Ag-source, the MSI pathway [29, 30] and genes associated with MSI status [31] have an impact on survival (based on hazard ratio in the human counterpart) (Table 1). Except for *Cope*, the theme of exclusive and distinct SNVs within the 328 and A7450 T1 M1 cell lines continues. However, its influence on survival remains elusive and has no impact, since the alterations are yet unknown or silent. Overall, cell line 328 shows a more substantial amount of affected genes associated with overall survival and disease-free survival. These are *Chmp5*, *Dhx32*, *Gadd45b*, and *Inadl*.

**Table 1:** SNVs in MLH1^−/−^ cell lines in tumor suppressor genes and potential association with survival

Gene	A7450 T1 M1	328	Survival		
*BAX*		NA		DFS	
*CHMP5*		MISSENSE	OS	DFS	DSS
*COPE*	NONE	SILENT	Unknown		
*DHX32*		SILENT	OS	DFS	
*DYNLT3*		NA		DFS	
*GADD45B*		NA	OS	DFS	DSS
*INADL*		NA	OS	DFS	DSS
*MTRF1*		NA		DFS	
*NME7*	NONE		OS	DFS	DSS
*RAC3*		SILENT	Unknown		
*SNRNP40*	NONE			DFS	
*SRP9*	NONE		Unknown		
*TMEM14C*	NONE		Unknown		

Based on COX p-value < 0.05

*OS* overall survival, *DFS* disease-free survival, *DSS* disease-specific survival

Then, the coding microsatellite (cMS) mutational profile was analyzed comparatively on a panel of putative MSI target genes (Table 2 and [18]). Overall, A7450 T1 M1 cells harbored mutations in half of the markers. The numbers of cMS mutations in 328 cells were lower (37%) and the genes affected differently, highlighting the individual profile even in these molecular closely matched Mlh1^−/−^ cells that harbor the very same germline mutation. Shared mutations were found in seven candidate genes, such as *Taf1b*, *Rfc3*, *Akt3*, and *Spen*. While these genes are all classified as tumor suppressors, they may have a high likelihood of being causative for this type of tumor. By deciphering the differences between these two samples in more detail, we identified some exclusive mutations in Mlh1^−/−^ A7450 T1 M1 cells whose resulting neo-Ags may have immunogenic potential. The most promising candidates, in this case, are *Senp6* and *Rasal2.* Consequently, we analyzed the frequency of spontaneous immune reactivity against the neoepitopes derived from a − 1 frameshift mutation in the cMS of these genes. However, in this test, no significant reactivity was detectable (*data not shown*), making these candidates unlikely to act as tumor rejection Ags.

**Table 2:** Mutational profile of MLH1^−/−^ derived cell lines using an in-house panel of cMS marker

cMS Marker	APC	Tmem60	Senp6	Phactr4	Aste1	Taf1b	Sdccag1	Rasal2	Lig4	Bend5	Supt16	C8a	Kcnma1	Rfc3	ERCC5	DNAJC2	IL1F9	Clock	Akt3	Spen	Frequency	
Sample/Repeat	A8	A8	A11	A10	A8	A8	A11	A8	A9	A8	A8	T8	A10	A10	A9	A8	A10	T9	T8	A8	(n)	(%)
328	wt	−1/−2	wt	wt	wt/−1	wt/−1	wt	wt	wt/−1	wt	wt/−1	wt	wt/−1	wt/−1	wt	−1	−1	wt/−1	wt/−1	wt/−1	12/32	37.50
7450 T1 M1	wt/−1	wt	wt/−1	−1	wt	wt/−1	−1	wt/−1	−1	wt/−1/−2	−1	MSI	wt/−1	wt/−1	wt/−1	wt	−1	wt	wt/−1	wt/−1	16/32	50.00

wt: wildtype; wt/−1: heterozygous mutation; −1: homozygous mutation

### Prolonged survival in the prophylactic setting

To test the immunogenicity of whole cancer vaccines on a more global level, Mlh1^−/−^ mice received two independent tumor lysates, either harboring high (= 328, 167 mutations/Mb) or moderate (= A7450 T1 M1, 27 mutations/Mb) TMB [21] (Fig. 1).

**Fig. 4:**
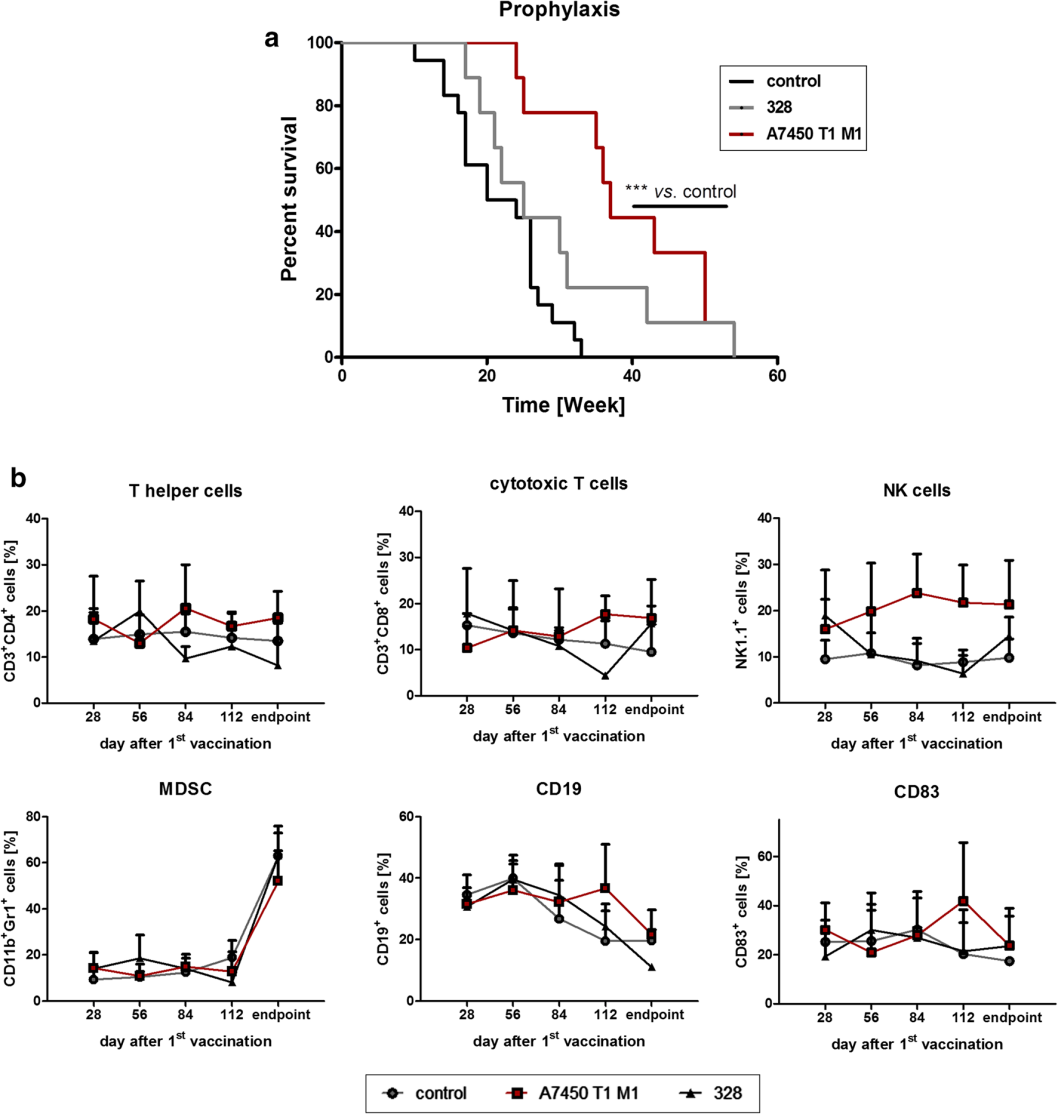
Prophylactic approach. Mice received repetitive local applications of the Mlh1^−/−^ A7450 T1 M1 and Mlh1^−/−^ 328 vaccine, respectively (10 mg/kg bw, s.c., n = 9 mice/group). Control mice were left untreated (n = 15 mice). **a** Kaplan–Meier survival curve analysis. *p < 0.001 A7450 T1 M1 vs. control; Log-rank (Mantel–Cox) test. **b** Immune phenotyping of peripheral blood was done before and during the course of vaccination until the experimental endpoint. Samples were analyzed by flow cytometry on a FACSVerse™, data analyses were performed using BD FACSuite software. Given are the % numbers of positive cells measured upon gating on viable cells

Prophylactic vaccination yielded significantly prolonged cancer-free survival in Mlh1^−/−^ A7450 T1 M1-treated mice. Median survival time was 37 weeks, whereas it was only 22 weeks in control mice (p < 0.001). The Mlh1^−/−^ 328 vaccine had a minor impact on survival, reaching a median survival of 25 weeks (Fig. 4a). The tumor spectrum observed in this study largely covers the distribution seen in Mlh1^−/−^ mice. Two thirds of Mlh1^−/−^ A7450 T1 M1-treated mice developed GIT or generalized lymphomas in the spleen; remaining mice developed lymphomas in the thymus (1 case), skin malignancies (1 case) or died spontaneously (2 cases). Mice receiving the Mlh1^−/−^ 328 tumor lysate showed a comparable tumor spectrum. Here, 70% suffered from GIT or generalized lymphomas in the spleen, one mouse developed a thymic lymphoma, and two mice died because of unknown malignancy (suspected lymphomagenesis).

The survival benefit of mice vaccinated with the Mlh1^−/−^ A7450 T1 M1 lysate was reflected by immunological changes in the peripheral blood. While T cell numbers only gradually increased, we observed elevated levels of circulating NK cells (Fig. 4b).

**Fig. 5:**
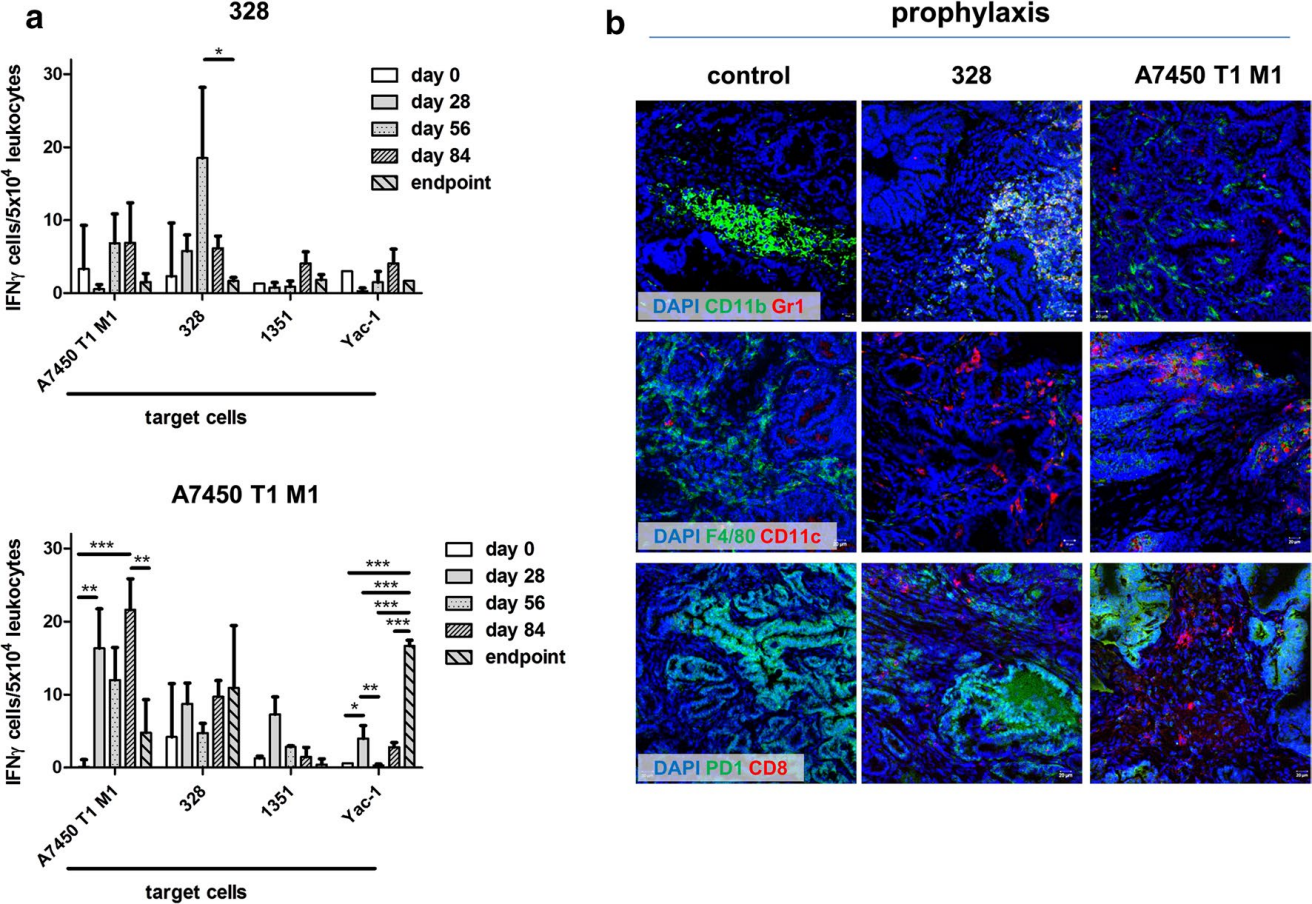
IFNγ ELISpot and tumor microenvironment. **a** Reactivity of peripheral blood leukocytes against Mlh1^−/−^ target cells as assessed during vaccination and at the experimental endpoint. The number of IFNγ secreting cells/5 × 10^4^ effector cells was determined after overnight co-incubation and quantification on an ELISpot reader as described in material and methods. *p < 0.05; **p < 0.01; ***p < 0.001 one-way ANOVA (Bonferroni multiple comparison). **b** Immunofluorescence was done on 4 µM slides stained with mAbs as stated in “Material and methods” section. Cell nuclei were stained with DAPI. Control tumors were highly infiltrated with CD11b^+^ granulocytes and F4/80^+^ macrophages. The infiltration pattern changed dependent on the vaccine. Analyses were done on a laser scanning microscope (Zeiss) using 20× objectives

Then, the reactivity of peripheral blood leukocytes was assessed upon co-incubation with different target cells by IFNγ-ELISpot assay (Fig. 5a). Autologous Mlh1^−/−^ tumor targets triggered IFNγ secretion of lymphocytes from vaccinated mice. The highest reactivity was seen between days 56 and 84 and mainly against target cells that were used for vaccination. We even observed differences between the two vaccines; A7450 T1 M1 cells evoked IFNγ secretion more effectively from lymphocytes than 328 cells (p < 0.01). In line with the increased number of NK cells upon A7450 T1 M1 vaccination, leukocytes from vaccinated mice reacted against NK target cells YAC-1 (p < 0.01).

### Tumor microenvironment

Next, the tumor microenvironment was studied in detail to explore the quantity and quality of leukocyte infiltrates. Prophylactic vaccination leveraged the microenvironment (Fig. 5b). The Mlh1^−/−^ A7450 T1 M1 vaccine largely prevented infiltration of CD11b^+^/Gr1^+^ myeloid-derived suppressor cells and F4/80^+^ tumor-associated macrophages (TAMs). While these cell types were barely detectable, we observed high numbers of infiltrating CD11c^+^ DCs as well as CD8^+^ cytotoxic T cells (CTL). By contrast, the Mlh1^−/−^ 328 lysate triggered MDSC infiltration in the tumor, CTL were occasionally found. PD1 expression was not altered by any vaccination and, thus, expression levels were highly comparable with control tumors.

### Therapeutic vaccination

**Fig. 6:**
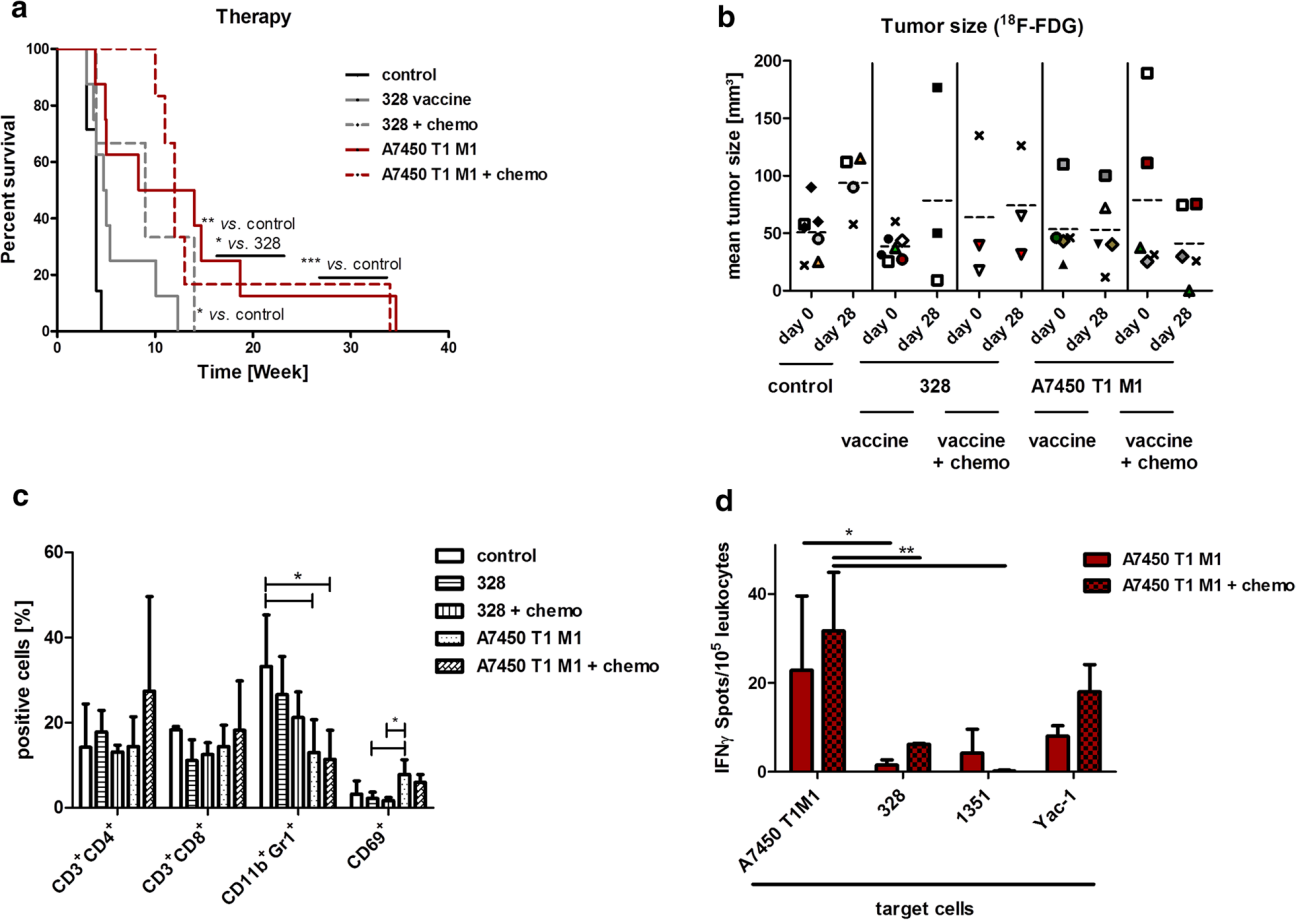
Therapeutic approach. Mice received repetitive local applications of cell line derived vaccines A7450 T1 M1 and 328, respectively (10 mg/kg bw, s.c., n = 8 mice/group). Control mice were left untreated (n = 7 mice). Combined chemo-vaccination was done by adding gemcitabine (100 mg/kg bw; Mlh1^−/−^ A7450 T1 M1: n = 6 and 328: n = 3 mice). **a** Kaplan–Meier survival curve analysis. *p < 0.05 328 + chemo vs. control; **p < 0.01 A7450 T1 M1 vs. control; *p < 0.05 A7450 T1 M1 vs. 328; Log-rank (Mantel–Cox) Test. **b** Mean tumor size determined by ^18^F-FDG PET/CT. In vivo imaging was done before and during vaccination. Tumors sizes were quantified using the inveon software. The symbols were standardized between day 0 and day 28, and each symbol is representative of one mouse (average tumor size in given resulting from the detected number of tumors/mouse). **c** Flow cytometry of splenic leukocytes. Phenotyping was done on splenocytes from control (n = 5) and vaccinated mice receiving monotherapy (328 and A7450 T1 M1, 3–5 mice/group) or combinations with gemcitabine (n = 3–5 mice/group). * p < 0.05; one-way ANOVA (Dunnett’s multiple comparison). **d** IFNγ ELISpot. Reactivity of splenocytes against MLH1^−/−^ target cells as assessed at the experimental endpoint. The number of IFNγ secreting cells/5 × 10^4^ effector cells was determined after overnight co-incubation and quantification on an ELISpot reader as described in material and methods. *p < 0.05; **p < 0.01; one-way ANOVA (Bonferroni multiple comparison)

Then, we moved to the therapeutic approach (Fig. 1). The survival benefit of mice treated with a lysate from the Mlh1^−/−^ A7450 cells compared to the 328 lysate was shown before [21] and (Fig. 6a). Here, the median overall survival was 11 weeks. By contrast, the 328 lysate failed to improve outcome, which was slightly longer than in untreated control mice (5 vs. 4 weeks). To see whether treatment can be improved by adding low-dose chemotherapy, the vaccination protocol was extended by gemcitabine given one day before treatment initiation, followed by 2 monthly injections (Figs. 1, 6a). With this combined chemo-vaccine, survival was prolonged in mice treated with cell-line derived tumor lysates 328 (9 weeks; p < 0.05 vs. control). With regard to the A7450 T1 M1+ chemo group, there was a trend towards longer progression-free survival, yet this did not reach statistical significance (hazard ratio: 0.9).

Accompanying PET/CT imaging largely reflected the survival data (Fig. 6b). 328-vaccinated tumors progressed, with no gross changes compared to untreated controls. Gemcitabine in conjunction with the lysate yielded stable disease. The same was true for tumors treated with the A7450 T1 M1 lysate, showing virtually no progression during the 1st weeks of treatment. Here again, combined chemo-immunotherapy improved tumor growth control. Individual tumors even tended to shrink (Fig. 6b). Still, the antitumoral stimulus provided by the combined chemo-vaccine was not strong enough to induce long-term regression, and tumors finally progressed.

### Immunological changes upon vaccination

Therapeutic vaccination altered splenic immune cell composition. Spleens from 328-vaccinated mice tended to have reduced amounts of CTL (Fig. 6c). Levels of CD11b^+^Gr1^+^ MDSC as well as CD69^+^ activated T cells remained similar to controls. Gemcitabine had no impact on immune cell distribution at all. Spleens from mice receiving the chemo-vaccine combinations had similar phenotypes as those treated with the 328 vaccine alone.

In contrast, the immune phenotype of spleens from A7450 T1 M1-vaccinated mice positively changed with significantly lower numbers of MDSCs but higher levels of activated CD69^+^ T cells (Fig. 6c). This effect was even independent of gemcitabine and thus related to the vaccine itself. Accompanying functional ELISpot analysis confirmed these findings with high reactivity against autologous target cells A7450 T1 M1 (Fig. 6d). Leukocytes from mice treated with the chemo-vaccine combination tended to have higher reactivity against NK cell targets YAC-1 compared to those getting the A7450 T1 M1 monotherapy. In line with the results from the prophylactic setting, there was no cross-reactivity against other Mlh1^−/−^ tumor targets, i.e. 328 and 1351.

**Fig. 7:**
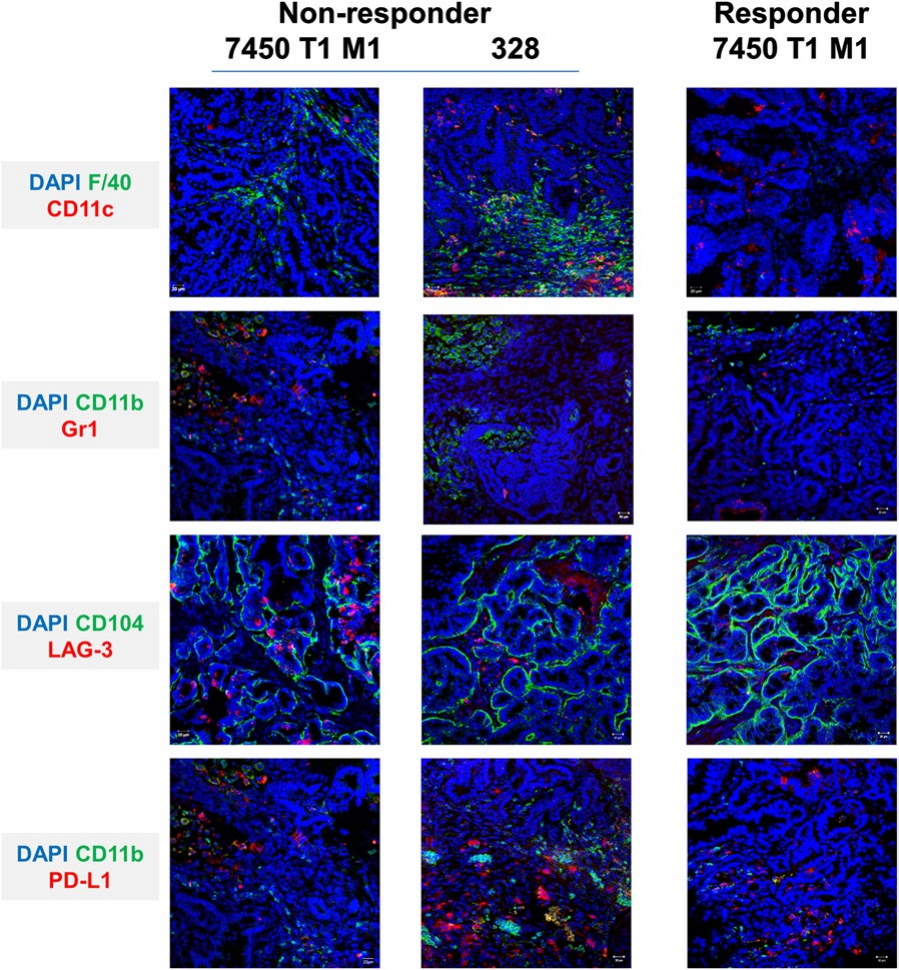
Tumor microenvironment. Immunofluorescence was done on 4 µM slides stained with mAbs as stated in the material and methods section. Cell nuclei were stained with DAPI. Representative images are given showing either GIT in which treatment failed (left panel) or, as in the case of A7450 T1 M1, treatment succeeded. Differences in leukocytic infiltration are evident. Analyses were done on a laser scanning microscope (Zeiss) using 20× objectives

We finally examined whether alterations were evident on tumor resection specimens in situ. Generally, A7450 T1 M1 vaccinated tumors were more infiltrated than 328-treated tumors (Fig. 7). By delineating mice that had no response from those achieving stable disease in PET/CT, we indeed found clear differences in the tumor microenvironment. Tumors of short-term survivors (328) were highly infiltrated with TAMs and had higher numbers of LAG-3- and PD-L1-expressing lymphocytes (Fig. 7). Granulocytes were rarely detectable. Resection specimens from long-term survivors harbored few TAMs, virtually no MDSCs or LAG-3^+^ lymphocytes. Hence, these data nicely reflect the in vivo response.

## Discussion

Here, we used the Mlh1^−/−^ mouse model and examined the protective value of two individual cancer vaccines made from autologous tumor cell cultures with different TMB [21]. The two cell lines, 328 and A7450 T1 M1, show exclusively distributed non-synonymous alterations in the pre-selected clinical hotspot regions. Every single amino acid appears exclusively, with *Arid1a* and *Apc*, being the only genes shared from both cell lines. This clearly shows that the tumors develop differently apart from the host, and with SNVs mainly affecting binding domains as well as occurring in tumor suppressors and interfering with the MSI status and/or the MSI signaling pathway.

By using cell lines for vaccination, this approach provides a virtually limitless source of neo-Ags, permits standardized, large-scale vaccine production, and is—from the economic point of view—very cost-effective [5]. To get an idea on the mutanome, whole-exome sequencing was performed on both cell lines in the very same passage later used for in vivo vaccination. Hypothetically, the number of neo-Ags correlates with immune activation and consequently, treatment outcome. However, we here provide evidence that the neo-Ag quality outranks quantity. By applying two vaccines that harbor the very same germline mutation, only one was able to activate T cells in vitro and mediated a survival benefit in the prophylactic situation. The 328 cell line was established directly from an ultra-hypermutated GIT with aggressive in situ growth behavior. Indeed, the mutations found in this cell line were mostly associated with a worse prognosis. The cell line A7450 T1 M1 was made from a moderately mutated GIT allografted in Mlh1^±^ mice that gave rise to stable in vitro growth [21]. While these two cell lines show no significant differences in growth kinetics, phenotype (MHC-I^+^, IDO^low^, PD-L1^+^) and drug response [19], their cytokine secretion profile greatly varies. A7450 T1 M1 cells secreted cytokines associated with favorable prognosis at least in colorectal cancer (such as GM-CSF, IL-1b) [32], with known ability to enhance NK cell activity and foster Th1 cell development. By contrast, the secretion profile of 328 cells nicely matched with a prototypic immunosuppressive cell line. The high inter-individual heterogeneity was further validated by additional mutational analysis that focused on cMS mutations, which are exclusive for MMR-D tumors. In fact, these two cell lines harbored only a few shared cMS mutations. By examining the spontaneous immune reactivity against selected neoepitopes, we, however, failed to observe significant reactivity, leaving the neo-Ags that confer immune responses unidentified. Still, the survival benefit of Mlh1^−/−^ A7450 T1 M1 vaccinated mice compared to those receiving the 328 lysate was reflected by systemic immunological changes. T cell numbers gradually increased, and mice had elevated levels of circulating NK cells, recognizing autologous target but also YAC-1 cells in ELISpot IFNγ assays. NK cells are a subset of innate lymphocytes with great potential to kill cancer cells directly, and thus, the elevated number of NK cells detected here may have also prevented early tumor formation [33–35]. Due to the sustained immunological pressure on (premalignant) tumor cells and the process of cancer editing, it is tempting to speculate that cancer cells either escaped NK cell control or directly induced loss of the NK cells’ cytotoxic ability. The latter was just recently shown in a preclinical breast cancer model and uncovers novel clues on how cancer cells escape NK cell surveillance [36]. Still, the vaccine itself stimulated both arms of the immune system. Though this immune activating stimulus was not strong enough to prevent tumor formation it altered the microenvironment, especially upon Mlh1^−/−^ A7450 T1 M1 vaccination. Here, numbers of tumor-infiltrating CD11c^+^ DCs were elevated; tumor-promoting MDSCs and TAMs low and provide a reasonable explanation for the delayed in vivo tumorigenesis compared to 328-vaccinated mice.

Therapeutic application prolonged progression-free survival, but again only when mice received the A7450 T1 M1 vaccine. The ultra-hypermutated-derived lysate 328 failed to provide a clear survival benefit. Adding chemotherapy to either vaccination improved the outcome by inducing long-term stable disease (≥ 4 weeks). Once more, tumor growth control was more effective in the A7450 T1 M1 combination group, assuming that the coupled application of the tumor lysate and low-dose chemotherapy induced immunogenic cell death. This converted dying cancer cells into a vaccine vulnerable to be taken up by DC. These, in turn, activated T cells to kill tumor targets. In support of this, half of the mice received partial remission. Here, chemotherapy itself may play a supportive role in re-activating the immune system against Mlh1^−/−^ tumors. Other preclinical studies likewise described boosted Ag cross-presentation, increased immune-supportive M1 macrophages, as well as circulating T cells upon gemcitabine [37, 38]. Indeed, spleens from treated mice in this study had higher numbers of activated T cells and significantly lower MDSC level. By unraveling the tumor microenvironment, differences became much more apparent, and tumors of long-term survivors had fewer immunosuppressive infiltrates (MDSCs, TAMs) than those that failed to respond. Whether responding tumors harbored less immunosuppressive infiltrates per se and were therefore better treatable by the given therapy or the applied regimen actively eliminated TAMs and MDSCs is a matter of speculation. Generally, the efficacy of cancer immunotherapy seems to be negatively correlated with MDSCs frequency and function [39, 40]. Still, a one-size-fits-all model does not exist. In pancreatic cancer patients, for instance, a high pre-vaccination MDSC value did not preclude an immune response [41], whereas higher MDSCs levels were associated with lower response rate in metastatic melanomas [42]. While most of these studies assessed pre-vaccination peripheral blood levels as biomarkers, the association between the attraction of immunosuppressive cells into the tumor and the development of secondary resistance to immunotherapy is yet unknown*.* Several extrinsic as well as intrinsic factors foster resistance, especially after initial response. Treatment failure might be finally attributable to insufficient T-cell responses (transient, low avidity, low magnitude); poor T-cell homing to Mlh1^−/−^ tumors, dysfunction or death of T cells within the tumor, and immune escape mediated by upregulation of immune-checkpoint molecules LAG-3 and PD-L1. Hence, the balance between immune-mediated tumor prevention/elimination and escape is a narrow ridge [43, 44]. A previous study identified several potential therapy-resistance genes, confirmed in CRISPR-based screens [45]. IFNγ—initially associated with tumor immunity also enhances the activation of the PD-1 signaling axis. Indeed, we also diagnosed higher numbers of LAG-3- and PD-L1-expressing lymphocytes in tumors of short-term survivors (mainly 328), while resections specimens from long-term survivors harbored virtually no LAG-3^+^ lymphocytes.

In humans, MMR-D tumors are often characterized by an increased density of intratumoral T cells and most patients are eligible to immunotherapy. Still, we here add evidence that the neo-Ag quality, rather than quantity defines response. These findings are supported by a recent study comparing pancreatic cancer and melanoma Ag load and T cell responses [46]. While the number of potential neo-Ags in pancreatic cancer samples was an order of magnitude lower than in melanoma, almost every tumor had a mutation that resulted in a predicted neo-Ag [47]. Comparable results were reported in hepatocellular carcinoma where the number of predicted neo-Ags did unexpectedly not correlate with effector and regulatory immune cell infiltration [48]. To discriminate immunogenic epitopes from a background set of mutated peptides, non-synonymous mutations should principally confer antitumoral vaccine activity. Hence, we tested the spontaneous immune response against a panel of putative immunogenic peptides. Still, in this setting no significant immune response was detectable, leaving the exact tumor rejection Ags unidentified.

To improve vaccine efficacy prospectively, some additional aspects must be considered: (I) the choice of the right target Ags, whose mutation frequency is high and ideally shared among cancers; (II) the time-interval and dosing of vaccines; (III) the route of application; (IV) the choice of adjuvant and/or combinatorial agent as well as (V) a change of the standard of care in humans from the tumor to the host by treating patients with immunotherapy in first-line and thus before a history of previous anti-cancer chemotherapy.

Finally, we would like to mention that there are some limitations to this study. Firstly, we only injected vaccines without additional adjuvants that might play a supporting role in immune stimulation. Secondly, mice were vaccinated with a lysate of only one cell line instead of different ones. Hence, there is a possibility that we have missed certain Ags that evoke immune responses when applied together and thus individual tumor clones may have been unrecognized.

## Conclusions

Prophylactic as well as therapeutic vaccination with whole tumor lysates delay tumor growth. Still, not only tumor mutational burden but also neoantigen quality predict vaccination efficacy. In addition to the number of mutations provided by a vaccine, the ability to evoke T cell responses and induce an inflamed tumor microenvironment is crucial for treatment responses.

## Data Availability

The datasets used and/or analyzed during the current study are available from the corresponding author on reasonable request.

## Abbreviations

Ag: Antigen
cMS: Coding microsatellite
DC: Dendritic cell
GIT: Gastrointestinal tumor
MDSC: Myeloid-derived suppressor cells
MMR-D: Mismatch repair deficiency
TAM: Tumor-associated macrophages
TMB: Tumor mutational burden
MSI: Microsatellite instability
